# Kaempferol Inhibits MMP-1-Mediated Migration and Invasion in Gemcitabine-Resistant Pancreatic Cancer Cells

**DOI:** 10.3390/nu18030380

**Published:** 2026-01-23

**Authors:** Saburo Sugita, Yoichi Matsuo, Masaki Ishida, Yuriko Uehara, Yuki Eguchi, Yuki Denda, Keisuke Nonoyama, Hiromichi Murase, Tomokatsu Kato, Kenta Saito, Takafumi Sato, Yushi Yamakawa, Hiroyuki Sagawa, Ryo Ogawa, Hiroki Takahashi, Akira Mitsui, Shuji Takiguchi

**Affiliations:** 1 Department of Gastroenterological Surgery, Graduate School of Medical Sciences, Nagoya City University, Nagoya 467-8601, Japan; ssabu3753@gmail.com (S.S.); i.masaki0103@gmail.com (M.I.); k.yuriko31@gmail.com (Y.U.); yukieg0802@gmail.com (Y.E.); denda@med.nagoya-cu.ac.jp (Y.D.); knonoyama0924@gmail.com (K.N.); muramen5.com@gmail.com (H.M.); tomo.k.g.w@gmail.com (T.K.); kentaxis777@gmail.com (K.S.); tsato.ncu@gmail.com (T.S.); uc19810116@gmail.com (Y.Y.); hiro18.hiroyuki.hiro@gmail.com (H.S.); ryogawancu@gmail.com (R.O.); coolsound1230@gmail.com (H.T.); a.mitsui.21@west-med.jp (A.M.); takiguch@med.nagoya-cu.ac.jp (S.T.); 2 Department of Gastroenterological Surgery, East Medical Center, Graduate School of Medical Sciences, Nagoya City University, Nagoya 464-8547, Japan

**Keywords:** pancreatic cancer, gemcitabine resistance, kaempferol, MMP-1, migration, invasion

## Abstract

**Background:** Pancreatic cancer (PaCa) has an extremely poor prognosis. This malignancy rapidly acquires resistance to gemcitabine (GEM), a key chemotherapeutic agent, yet the mechanisms underlying this resistance remain incompletely understood. We previously established GEM-resistant (GEM-R) PaCa cell lines and found that these cells exhibit constitutively increased levels of matrix metalloproteinase-1 (MMP-1), which contributes to the invasion and metastasis of PaCa. Kaempferol, a naturally occurring flavonoid found in many plant species, has been shown to exhibit antitumor effects across a range of cancers. **Methods/Results:** This study demonstrated that non-cytotoxic concentrations of kaempferol significantly decrease MMP-1 protein expression in GEM-R PaCa and suppress their migration and invasion capacities. Western blot analysis demonstrated that MMP-1 protein levels were upregulated in GEM-R PaCa cells and decreased upon kaempferol exposure. In Transwell migration/invasion and wound healing assays, GEM-R PaCa cell lines exhibited enhanced migration and invasion capacities compared with GEM-S cells, whereas kaempferol treatment suppressed these properties, similar to the effects observed by MMP-1 knockdown or treatment with the MMP inhibitor batimastat. Furthermore, kaempferol treatment reduced phosphorylated Akt expression and NF-κB p65 activity. **Conclusions:** These findings indicate that kaempferol suppresses the migratory and invasive abilities of PaCa cells by downregulating MMP-1 through negative regulation of the Akt and NF-κB signaling cascades, while kaempferol holds promise as a treatment strategy for GEM-R PaCa.

## 1. Introduction

Pancreatic cancer (PaCa) continues to pose a significant clinical challenge due to its exceptionally high lethality. In the U.S., patients diagnosed with PaCa have a median survival of around 4 months, and only 13% survive beyond 5 years. At present, pancreatic cancer ranks as the third most common cause of cancer mortality, and projections indicate this may rise to second place by 2030 [1]. In the United States, the estimated figures for 2024 include 66,400 newly diagnosed cases and 51,750 fatalities [2]. Due to its asymptomatic and aggressive nature, PaCa is often diagnosed at an advanced, incurable stage; thus, chemotherapy remains a cornerstone of treatment.

Gemcitabine (GEM) is a standard chemotherapeutic agent for PaCa; however, GEM resistance commonly develops early during therapy. Reported mechanisms of GEM resistance include reduced GEM uptake, increased detoxification, and elevated levels of endogenous substrates competing with GEM activation. Our previous work demonstrated that multidrug resistance-associated protein 1 (MRP1), a transporter that effluxes anticancer drugs, is upregulated in GEM-resistant (GEM-R) PaCa cells [3]. Despite these findings, the mechanisms underlying GEM-R are still not fully elucidated, and new therapeutic strategies are urgently needed.

Matrix metalloproteinases (MMPs), a group of zinc-dependent endopeptidases, play a key role in remodeling the extracellular matrix and are categorized into collagenases, gelatinases, stromelysins, and matrilysins [4,5]. MMPs utilize zinc ions as catalytic cofactors for their proteolytic activity and are structurally stabilized by calcium ions [6]. MMPs promote tumor invasion and metastasis by degrading extracellular matrix components and regulate the signaling pathways involved in inflammation, angiogenesis, and cellular proliferation [7]. MMP-1, the first identified MMP [8], plays a key role in collagen degradation and cancer cell invasion. Although MMPs have long been implicated in tumor progression, no effective MMP-targeted therapy has yet reached clinical application.

We previously developed GEM-R PaCa cell lines [3,9] and observed consistently elevated MMP-1 expression in these cells. Analysis of patient datasets using the Kaplan–Meier Plotter (Pan-cancer RNAseq, TCGA) further showed that high MMP-1 expression is associated with a poor prognosis in PaCa. These findings highlight MMP-1 as a candidate for therapeutic intervention in GEM-R PaCa.

Kaempferol, a naturally occurring flavonoid abundant in fruits, vegetables, and conventionally used medicinal herbs [10], has attracted increasing attention due to its anti-inflammatory and anticancer properties. Its antitumor effects have been reported in various malignancies, including PaCa. In colorectal cancer, kaempferol has been shown to suppress MMP-1 expression [4]; however, whether kaempferol regulates MMP-1 in PaCa, particularly in GEM-R cells, remains unknown.

This research was conducted to examine MMP-1 expression and the enhanced migration/invasion characteristics associated with GEM resistance, and to investigate the MMP-1 inhibitory potential of kaempferol and cell motility in GEM-R PaCa cell lines, comparing its effects with those of the MMP inhibitor batimastat and MMP-1 knockdown.

## 2. Materials and Methods

### 2.1. Reagents

Kaempferol was obtained from Santa Cruz Biotechnology (cat. no. sc-202679B; Dallas, TX, USA) and batimastat from Selleck Chemicals (cat. no. S7155; Houston, TX, USA). Solutions of kaempferol and batimastat were individually prepared using dimethyl sulfoxide (Sigma-Aldrich, St. Louis, MO, USA) as the solvent. Recombinant human interleukin-1β (IL-1β) was purchased from PeproTech Inc. (cat. no. 200-01B-100UG; Cranbury, NJ, USA) and dissolved in distilled water.

### 2.2. Cell Lines and Cell Culture

The human pancreatic duct epithelial (HPDE) cell line H6c7 (cat. no. ECA001-FP; Kerafast, Newark, CA, USA) was cultured using keratinocyte serum-free medium (Gibco/Thermo Fisher Scientific, Rockford, IL, USA). Human pancreatic ductal adenocarcinoma cell lines (AsPC-1, MIA PaCa-2, PANC-1, and SW1990) were obtained from ATCC (Manassas, VA, USA). AsPC-1 cells were cultured in RPMI-1640 medium (Sigma Aldrich; Merck KgaA, Darmstadt, Germany), while the other pancreatic ductal adenocarcinoma cell lines were maintained in Dulbecco’s Modified Eagle Medium (Sigma Aldrich; Merck KGaA).

Unless otherwise specified, all media contained 10% fetal bovine serum (FBS; Gibco/Thermo Fisher Scientific), 100 U/mL penicillin, 100 µg/mL streptomycin, and 25 µg/mL amphotericin B. All cell lines were incubated at 37 °C in a humidified atmosphere with 5% CO_2_.

### 2.3. Establishment of GEM-R PaCa Cell Lines

GEM-R PaCa cell lines were established as described previously [3,9]. Briefly, parental PaCa cell lines (AsPC-1, MIA PaCa-2, PANC-1, and SW1990) were treated with increasing concentrations of GEM (Eli Lilly Japan K.K., Kanagawa, Japan) followed by repeated passaging.

The half-maximal inhibitory concentration (IC_50_) of GEM in each cell line was first determined using the WST-1 assay (cat. no. MK400; Takara Bio, Yamanashi, Japan). Cells were then exposed to GEM at their respective IC_50_ concentrations, and the IC_50_ was re-evaluated after each passage. Cell lines with a GEM IC_50_ value more than 50-fold higher than that of the parental cells were defined as GEM-R. The WST-1 assay was performed using six samples per concentration for each cell line.

### 2.4. Cytotoxicity Assay

The cytotoxicity of kaempferol and batimastat was assessed using the WST-1 assay. GEM-sensitive (GEM-S) and GEM-R MIA PaCa-2 or SW1990 cells were seeded at 3 × 10^3^/well in 96-well plates (100 µL/well). Cells were treated for 24 h with kaempferol (0–250 µM) or batimastat (0–4000 ng/mL). The WST-1 assay was performed using six samples per concentration for each cell line. The absorbance was measured using the SpectraMax ABS microplate reader (Molecular Devices, San Jose, CA, USA) to determine cell viability.

### 2.5. siRNA Transfection

siRNA targeting MMP-1 (siMMP-1; cat. no. AM16708) and negative control siRNA (siNC; cat. no. 4390843) were purchased from Thermo Fisher Scientific. PaCa cells were plated in 6-well culture dishes and allowed to grow until reaching 70–80% confluency without antibiotics. Following the guidelines provided by the manufacturer, 100 nM siRNA was transfected into cells using Lipofectamine RNAiMAX in Opti-MEM medium (Thermo Fisher Scientific) without antibiotics or FBS at 37 °C for 24 h. After transfection, the cells were used for protein extraction, Transwell assays, and wound healing assays.

### 2.6. Western Blotting

Proteins were extracted using RIPA Lysis and Extraction Buffer (cat. no. 89900; Thermo Fisher Scientific), with added protease and phosphatase inhibitor mixtures. Protein levels were quantified via the Pierce BCA assay kit. The protein samples (30 µg) were denatured at 90 °C for 5 min, separated on 10% Mini-PROTEAN TGX precast gels (Bio-Rad Laboratories, Inc., Hayward, CA, USA), and then transferred to nitrocellulose membranes. Blocking and antibody incubation were performed using the iBind Flex Western System (Thermo Fisher Scientific).

The primary antibodies included anti-MMP-1 (1:500; cat. no. 54376; Cell Signaling Technology, Danvers, MA, USA), anti-GAPDH (1:2000; cat. no. 2118, Cell Signaling Technology), anti-Akt (1:1000; cat. no. 4691), and anti-phospho-Akt (1:2000; cat. no. 8200, Cell Signaling Technology). Horseradish peroxidase (HRP)-conjugated goat anti-rabbit secondary antibody (cat. no. P0448; DAKO/Agilent, Santa Clasa, CA, USA) was used at the appropriate dilution. The protein signals were visualized using SuperSignal West Pico PLUS or Pierce ECL substrates (Thermo Fisher Scientific) and detected using the Amersham Imager 600 (Cytiva, Uppsala, Sweden). Band intensities were quantified using ImageJ software (version 1.52v). For evaluation of MMP-1 expression and Akt phosphorylation, kaempferol and batimastat, and IL-1β were administered for 24 h. To assess the effects on Akt phosphorylation, kaempferol was administered for 24 h and IL-1β for 1 h. Band density was measured three times for each sample. Original uncropped blots are provided where available.

### 2.7. Transwell Migration and Invasion Assays

Transwell assays were performed using the Boyden chamber method. Falcon 8.0 µm pore inserts were used in the migration assays, and Corning BioCoat Matrigel invasion chambers in the invasion assays. The upper chambers contained 500 µL serum-free medium, and the lower chambers 750 µL medium supplemented with 10% FBS. PaCa cells (1 × 10^5^) were placed in the upper chamber and allowed to incubate at 37 °C for 24 h. Cells that had migrated/invaded to the lower surface were stained with Diff-Quick, and the cells in nine random fields (×200) were imaged and counted.

### 2.8. Wound Healing Assay

GEM-S and GEM-R MIA PaCa-2 or SW1990 cells (1.0 × 10^5^) were plated in 6-well plates and cultured until they reached at least 90% confluency. A straight wound was generated on the cell monolayer using a sterile P10 pipette tip. Images were taken immediately (0 h) using the BZ-X710 microscope (Keyence, Osaka, Japan). After imaging, the cells were cultured in FBS-free medium containing kaempferol, batimastat, or IL-1β. The same fields were imaged at 24 h, and the ImageJ software was used to measure the wound area. Nine wound areas were measured, and the rate of wound healing was determined using the following formula:

Closure rate (%) = (S_0_h − S_24_h)/S_0_h × 100

### 2.9. Preparation of Nuclear Extracts and Measurement of NF-κB p65 Activity

Extraction of nuclear protein and NF-κB p65 activity evaluation were performed as described previously. GEM-S and GEM-R MIA PaCa-2 and SW1990 cells were grown to 80–90% confluence, after which they were treated with kaempferol for 2 h and with IL-1β for 30 min. After treatment, nuclear protein extraction was carried out with a commercially available Nuclear Extraction Kit (Active Motif, Carlsbad, CA, USA). Quantification of protein concentration was performed using the BCA assay. The TransAM NF-κB p65/p50 Transcription Factor Assay Kit (cat. no. 40096; Active Motif) was used to quantify NF-κB p65 activity. Measurements were performed in quadruplicate, and values are shown as fold changes normalized to the GEM-S control.

### 2.10. Statistical Analysis

All values are reported as the mean ± standard deviation. Statistical analyses were carried out with EZR v. 1.41 (Saitama Medical Center, Jichi Medical University, Japan). Data are presented as the mean ± standard deviation. Unpaired *t*-tests were applied for comparisons between two groups. For analyses involving three or more groups, one-way analysis of variance (ANOVA) with Bonferroni correction was performed. Results were considered statistically significant at *p* < 0.05.

## 3. Results

### 3.1. IC_50_ Values of GEM in PaCa Cell Lines

**Figure 1:**
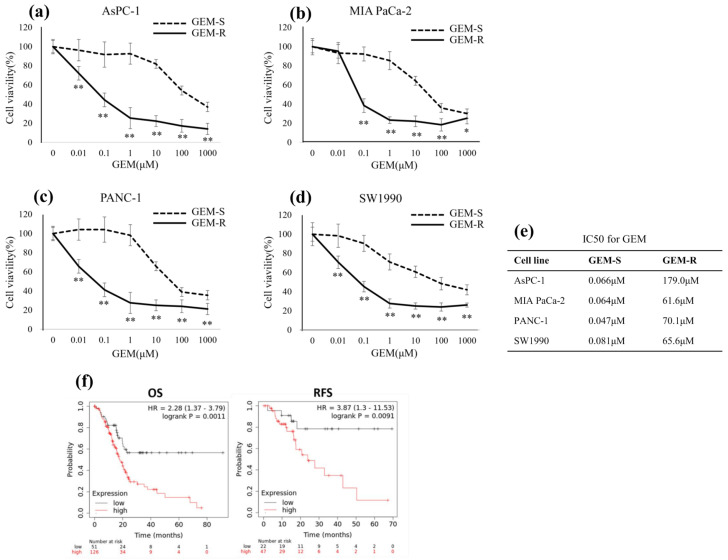
Effect of GEM on the proliferation of GEM-S and GEM-R PaCa cell lines. (**a**–**d**) GEM-S and GEM-R cells of each PaCa cell line (AsPC-1, MIA PaCa-2, PANC-1, and SW1990) were seeded into 96-well plates and treated with GEM at the indicated concentrations for 48 h. Cell proliferation was measured using a WST-1 assay (*n* = 6). Values are expressed as the mean ± SD. * *p* < 0.05; ** *p* < 0.01. (**e**) IC_50_ values for GEM were determined for each PaCa cell line. Comparisons between the GEM-S and GEM-R groups were assessed using an unpaired Student’s *t*-test. (**f**) Kaplan–Meier curves showing OS (177 patients) and RFS (69 patients) of PaCa patients obtained from the Kaplan–Meier Plotter database.

WST-1 assays were performed to determine the IC_50_ values of GEM in the established GEM-R PaCa cell lines (AsPC-1, MIA PaCa-2, PANC-1, and SW1990). After treatment with various concentrations of GEM for 48 h, cell viability was assessed using the WST-1 assay. The IC_50_ values after 48 h of GEM treatment were as follows: 0.066 and 179 µM for GEM-S and GEM-R AsPC-1 cells; 0.64 and 61.6 µM for GEM-S and GEM-R MIA PaCa-2 cells; 0.047 and 67.6 µM for GEM-S and GEM-R PANC-1 cells; and 0.081 and 95.6 µM for GEM-S and GEM-R SW1990 cells, respectively (Figure 1a–e).

### 3.2. MMP-1 Protein Expression in the PaCa Cell Lines

**Figure 2:**
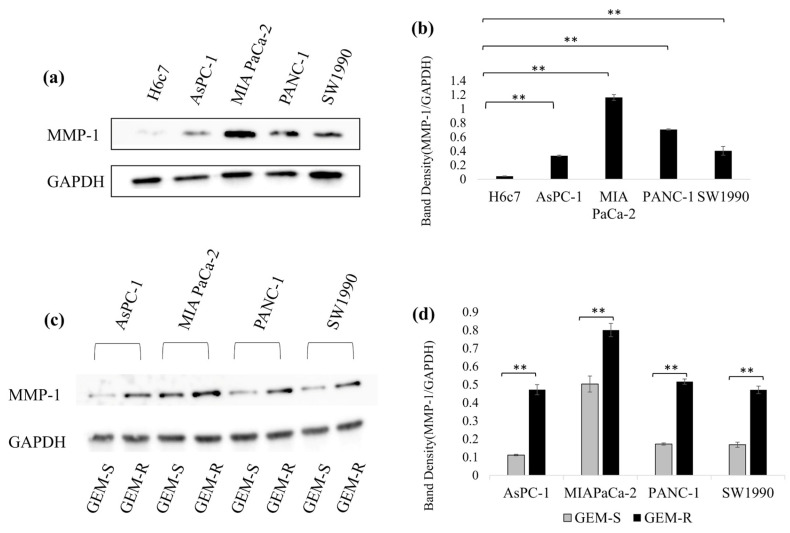
(**a**,**b**) MMP-1 protein expression in GEM-S PaCa cells. MMP-1 protein levels in the HPDE cell line (H6c7) and GEM-S PaCa cell lines (AsPC-1, MIA PaCa-2, PANC-1, and SW1990) were examined by Western blotting. Band densities were normalized to GAPDH. Comparisons were performed using one-way ANOVA. Data are presented as the mean ± SD. ** *p* < 0.01. (**c**,**d**) MMP-1 protein expression in GEM-R PaCa cells. MMP-1 expression in each GEM-R PaCa cell line was compared with its GEM-S counterpart using an unpaired Student’s *t*-test. Data are presented as the mean ± SD. ** *p* < 0.01.

The protein levels of MMP-1 in the HPDE cell line (H6c7) and PaCa cell lines (AsPC-1, MIA PaCa-2, PANC-1, and SW1990) were evaluated by Western blotting. All GEM-S PaCa cell lines showed significantly higher MMP-1 expression compared with the H6c7 cell line (*p* = 0.000001, 3.23 × 10^−10^, 0.00000003, 0.0006) (Figure 2a,b).

Furthermore, MMP-1 expression was markedly increased in all GEM-R PaCa cell lines compared with their corresponding GEM-S counterparts. Specifically, the MMP-1 level in GEM-R AsPC-1, GEM-R MIA PaCa-2, GEM-R PANC-1, and GEM-R SW1990 cells was elevated by 322% (*p* = 0.00003), 59% (*p* = 0.0008), 200% (*p* = 0.00003), and 179% (*p* = 0.00003), respectively, compared with the respective parental GEM-S cell lines (Figure 2c,d).

### 3.3. IC_50_ Values of Kaempferol and Batimastat in PaCa Cell Lines

**Figure 3:**
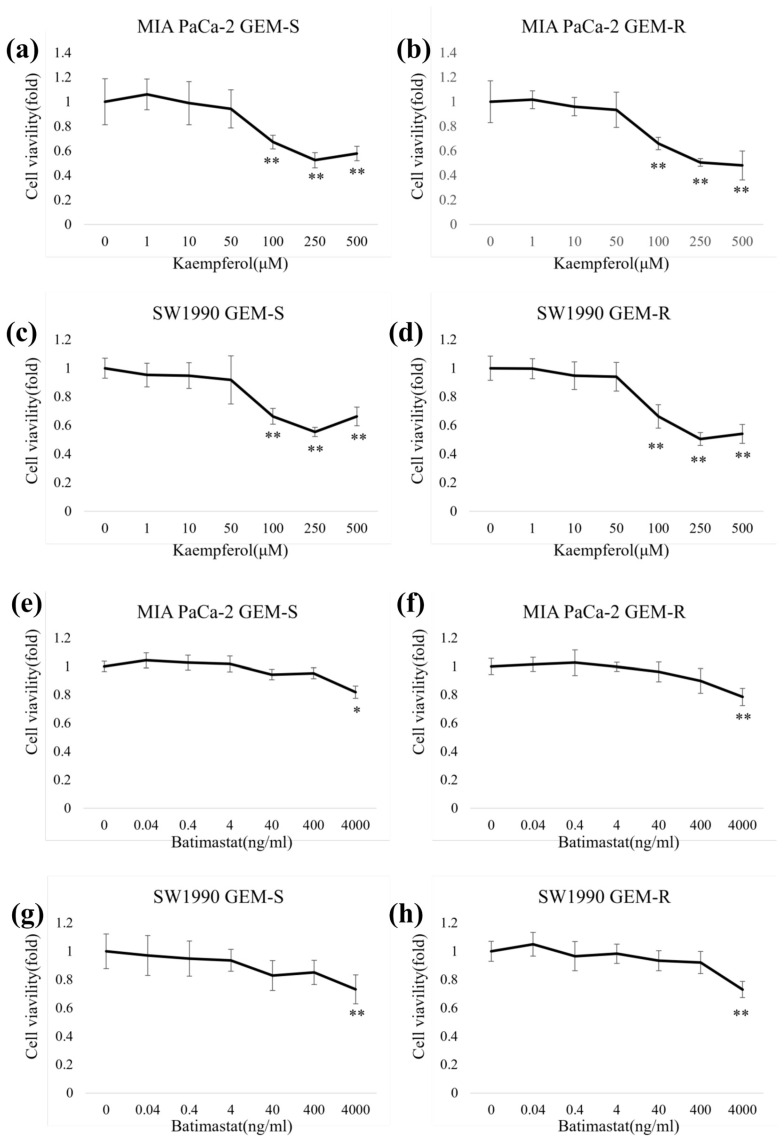
Cytotoxicity of kaempferol (**a**–**d**) and batimastat (**e**–**h**) in PaCa cell lines. GEM-S and GEM-R cells (MIA PaCa-2 and SW1990) were seeded into 96-well plates and treated with kaempferol or batimastat at the indicated concentrations for 24 h. Cell proliferation was measured using a WST-1 assay. Comparisons with control were assessed using an unpaired Student’s *t*-test. Data are presented as the mean ± SD. * *p* < 0.05. ** *p* < 0.01.

Before performing subsequent experiments, we assessed the cytotoxicity of kaempferol and batimastat using the WST-1 assay. After 24 h of treatment, no significant cytotoxicity of kaempferol or batimastat was observed at concentrations up to 50 µM (Figure 3a–d) or 400 ng/mL, respectively (Figure 3e–h). Based on these findings, 50 µM kaempferol and 400 ng/mL batimastat were selected as the concentrations to use in subsequent experiments to avoid cytotoxic effects.

### 3.4. Effect of Kaempferol Treatment on MMP-1 Protein Expression in PaCa Cell Lines

**Figure 4:**
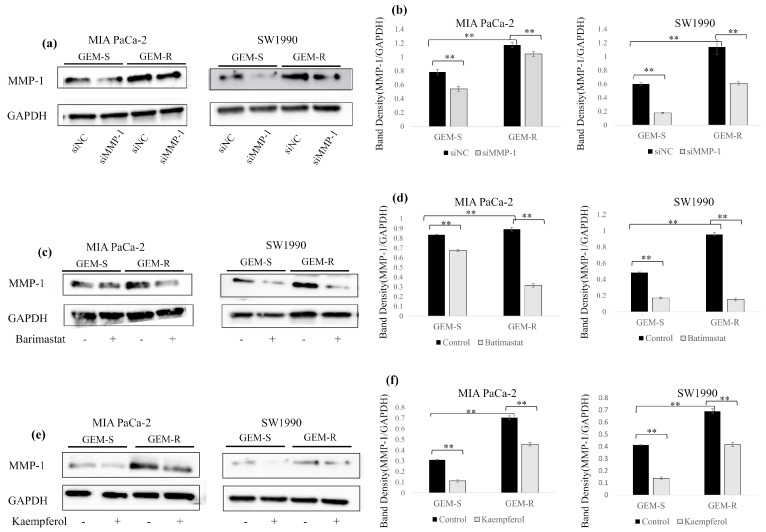
(**a**,**c**,**e**) Changes in MMP-1 expression following MMP-1 knockdown or treatment with kaempferol or batimastat, examined by Western blotting. (**b**,**d**,**f**) Band densities were normalized to GAPDH. Comparisons between groups were assessed using one-way ANOVA followed by Bonferroni’s test. Data are presented as the mean ± SD. ** *p* < 0.01.

We evaluated the effects of MMP-1 knockdown, 400 ng/mL batimastat, and 50 µM kaempferol on MMP-1 protein expression using Western blotting. In both GEM-S and GEM-R MIA PaCa-2 cells, MMP-1 expression was 31.1% and 11.0% lower in cells transfected with siMMP-1 than in those transfected with siNC, respectively. Similarly, in GEM-S and GEM-R SW1990 cells, MMP-1 expression was 70.3% (*p* = 0.0001) and 46.7% (*p* = 0.00002) lower in siMMP-1-transfected than siNC-transfected cells, respectively (Figure 4a,b).

Batimastat reduced MMP-1 protein levels in all PaCa cell lines. In GEM-S and GEM-R MIA PaCa-2 cells, MMP-1 expressions were 19.4% and 64.5% lower compared with the control group, respectively. In GEM-S and GEM-R SW1990 cells, batimastat reduced MMP-1 expression by 64.7% (*p* = 0.00000001) and 83.9% (*p* = 0.00002), respectively (Figure 4c,d).

Kaempferol also reduced MMP-1 expression in all cell lines examined. Kaempferol decreased MMP-1 expression by 63.8% and 35.2% in GEM-S and GEM-R MIA PaCa-2 cells, respectively, and by 66.8% (*p* = 0.00001) and 39.6% (*p* = 0.000005) in GEM-S and GEM-R SW1990 cells, respectively (Figure 4e,f).

### 3.5. Effect of GEM-R and MMP-1 Knockdown on the Migration of PaCa Cells

Cell migration following MMP-1 knockdown was evaluated by Transwell migration and wound healing assays. In both MIA PaCa-2 and SW1990 cell lines, GEM-R cells exhibited increased migration ability compared with GEM-S cells (*p* = 6.8 × 10^−10^, 0.01), and MMP-1 knockdown significantly suppressed this enhancement.

**Figure 5:**
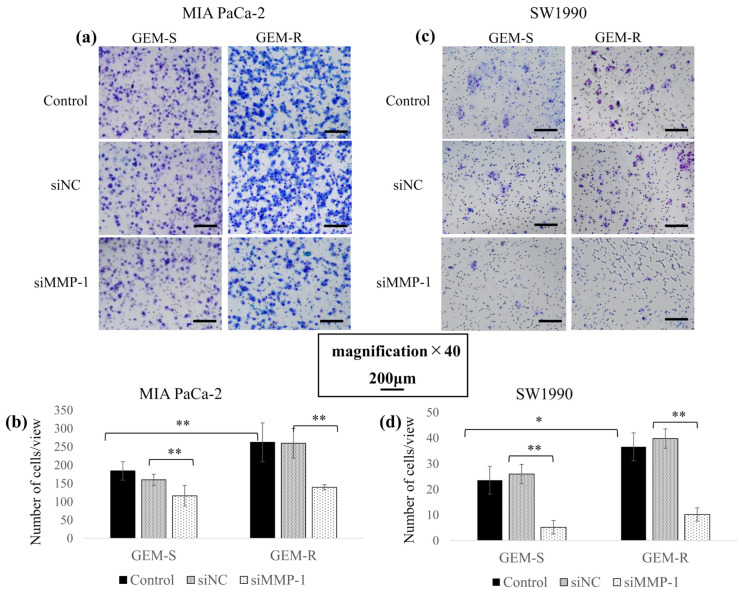
Effects of MMP-1 knockdown on migratory ability assessed by Transwell migration assay. After MMP-1 knockdown, GEM-S and GEM-R MIA PaCa-2 and SW1990 cells (1 × 10^5^) were seeded in uncoated upper chambers and cultured for 24 h. Migrated cells on the lower surface were fixed, stained, and imaged (**a**,**c**). Magnification: ×40; scale bar: 200 μm. Nine random fields were imaged, and migrated cells were quantified (**b**,**d**). Comparisons were assessed using one-way ANOVA followed by Bonferroni’s test. Data are presented as the mean ± SD. * *p* < 0.05; ** *p* < 0.01.

In the Transwell migration assay, following MMP-1 knockdown, the number of migrating cells was reduced by 27.9% (*p* = 0.03) and 46.3% (*p* = 1.1 × 10^−10^) in GEM-S and GEM-R MIA PaCa-2 cells, respectively, and by 77.9% (*p* = 8.2 × 10^−6^) and 72.1% (*p* = 1.5 × 10^−9^) among SW1990 GEM-S and GEM-R cells, respectively, compared with the siNC-transfected cells (Figure 5a–d).

**Figure 6:**
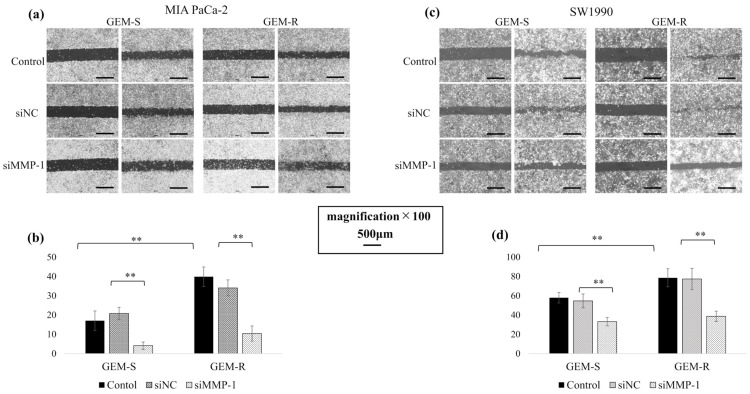
Inhibitory effects of MMP-1 knockdown on migration assessed by wound-healing assay. (**a**,**b**) GEM-S and GEM-R PaCa cells (1–2 × 10^5^) were seeded into 6-well plates and treated with siNC or siMMP-1. When semi-confluent, a straight wound was created with a sterile P10 tip. Cells were incubated in 0% FBS medium for 24 h and imaged (*n* = 9). Magnification: ×100; scale bar: 500 μm. (**c**,**d**) Wound areas were measured using ImageJ, and wound closure (%) was calculated: % wound area filled = (S_0_ − S_24_)/S_0_ × 100. Comparisons were assessed using one-way ANOVA followed by Bonferroni’s test. Data are presented as the mean ± SD. ** *p* < 0.01.

Consistent results were observed in the wound healing assay. MMP-1 knockdown reduced relative wound closure in both cell lines, indicating suppressed migration. The wound closure rate decreased from 20.8% to 4.14% and from 34.0% to 10.4% in GEM-S and GEM-R MIA PaCa-2 cells, respectively, and from 54.6% to 33.1% and from 78.6% to 38.7% in GEM-S and GEM-R SW1990 cells, respectively, compared with siNC-transfected cells (Figure 6a–d).

### 3.6. Effect of Kaempferol and Batimastat on PaCa Cell Migration, Including IL-1β-Induced Migration

**Figure 7:**
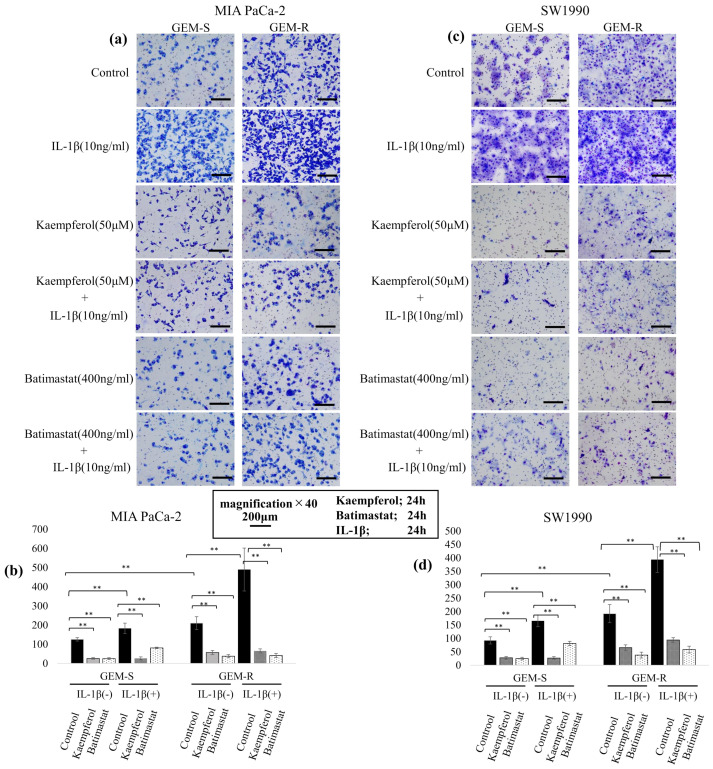
Effects of IL-1β, kaempferol, and batimastat on migration assessed by Transwell assay. PaCa cells (1 × 10^5^) were treated with IL-1β, kaempferol, or batimastat for 24 h and subjected to migration assays similar to Figure 5. Migrated cells were fixed, stained, and imaged (**a**,**c**). Magnification: ×40; scale bar: 200 μm. Quantification was performed from nine random fields (**b**,**d**). Comparisons were assessed using one-way ANOVA with Bonferroni’s test. Data are presented as the mean ± SD. ** *p* < 0.01.

To examine the effects of kaempferol and batimastat on PaCa cell migration, Transwell migration and wound healing assays were conducted. Both agents suppressed the migration of PaCa cell lines, including that enhanced by IL-1β. In the Transwell migration assay, 50 µM kaempferol and 400 ng/mL batimastat significantly reduced the number of migrating cells among both GEM-S and GEM-R MIA PaCa-2 and SW1990 cells. IL-1β increased the number of migrating cells among all PaCa cell lines; however, this increase was inhibited by both kaempferol and batimastat. Kaempferol reduced the migrating cell numbers by 79.1% and 72.9% (*p* = 1.7 × 10^−7^ and 1.2 × 10^−12^) and batimastat by 81.3% and 82.0% (*p* = 1.1 × 10^−7^, 4.3 × 10^−15^) among GEM-S and GEM-R MIA PaCa-2 cells, respectively. Among GEM-S and GEM-R SW1990 cells, kaempferol reduced migration by 70.1% and 66.0% (*p* = 1.8 × 10^−9^ and 1.2 × 10^−12^) and batimastat by 73.2% and 80.3% (*p* = 1.1 × 10^−7^ and 4.3 × 10^−15^), respectively. Kaempferol and batimastat also suppressed IL-1β-induced migration. Kaempferol reduced the number of IL-1β-induced migrating cells by 70.1% and 65.9% (*p* = 8.5 × 10^−12^ and *p* < 2 × 10^−16^) among GEM-S and GEM-R MIA PaCa-2 cells and by 83.9% and 76.2% (both *p* < 2 × 10^−16^) among GEM-S and GEM-R SW1990 cells, respectively, while batimastat reduced these numbers by 51.0% and 85.2% (both *p* < 2 × 10^−16^) among GEM-S and GEM-R SW1990 cells, respectively (Figure 7a–d). The inhibitory effect of kaempferol on cell migration was also confirmed in GEM-S and GEM-R AsPC-1 and PANC-1 cell lines. 50 µM kaempferol significantly reduced the number of migrating cells among both GEM-S and GEM-R AsPC-1 and PANC-1 cells. Kaempferol reduced the migrating cell numbers by 51.6% and 46.7% (*p* = 1.5 × 10^−5^ and 1.6 × 10^−12^) among GEM-S and GEM-R AsPC-1 cells, respectively. Among GEM-S and GEM-R PANC-1 cells, kaempferol reduced migration by 70.1% and 66.0% (both *p* < 2 × 10^−16^), respectively (see Supplementary Materials, Figure S1).

**Figure 8:**
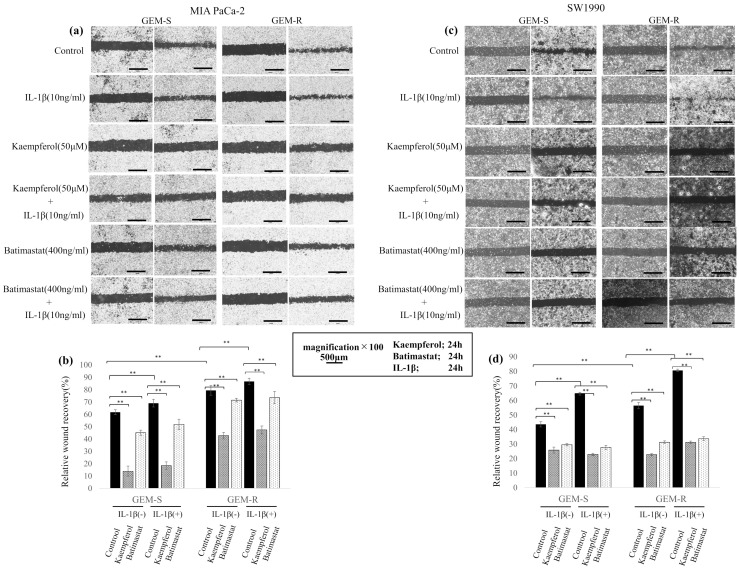
Effects of IL-1β, kaempferol, and batimastat on migration assessed by wound-healing assay. Procedures were performed as in Figure 6. After reaching semi-confluence, cells were treated with IL-1β, kaempferol, or batimastat for 24 h, and a linear wound was made. After 24 h in serum-free medium, wound closure was imaged (*n* = 9) (**a**,**c**). Magnification: ×100; scale bar: 500 μm. Relative wound recovery was quantified as described in Figure 6 (**b**,**d**). Comparisons were assessed using one-way ANOVA with Bonferroni’s test. Data are presented as the mean ± SD. ** *p* < 0.01.

In the wound healing assay, suppression of migration by kaempferol and batimastat was also observed. Kaempferol and batimastat reduced the wound closure rate from 61.8% to 13.7% and 45.1% (both *p* < 2 × 10^−16^) and from 79.5% to 42.7% and 71.6% (both *p* < 2 × 10^−16^) in GEM-S and GEM-R MIA PaCa-2 cells, respectively, and from 43.6% to 25.9% and 29.6% (both *p* < 2 × 10^−16^) and from 56.5% to 22.8% and 31.3% (both *p* < 2 × 10^−16^) in GEM-S and GEM-R SW1990 cells, respectively. Kaempferol and batimastat also suppressed IL-1β-induced wound closure from 69.1% to 18.4% and 51.9% (*p* < 2 × 10^−16^, *p* = 5.9 × 10^−11^) and from 86.7% to 47.4% and 73.6% (*p* = 7.6 × 10^−7^, 9 × 10^−9^) in GEM-S and GEM-R MIA PaCa-2 cells, respectively, and from 64.7% to 22.9% and 27.7% (both *p* < 2 × 10^−16^) and from 80.6% to 31.2% and 33.7% (*p* < 2 × 10^−16^) in GEM-S and GEM-R SW1990 cells, respectively (Figure 8a–d).

These findings suggest that kaempferol effectively suppresses both the baseline and IL-1β-enhanced migration of PaCa cells, comparable with the effect of the MMP inhibitor batimastat.

The inhibitory effect of kaempferol on cell migration was also confirmed in GEM-S and GEM-R AsPC-1 and PANC-1 cell lines. 50 µM kaempferol significantly reduced the number of migrating cells among both GEM-S and GEM-R AsPC-1 and PANC-1 cells. Kaempferol reduced the migrating cell numbers by 51.6% and 46.7% (*p* = 1.5 × 10^−5^ and 1.6 × 10^−12^) among GEM-S and GEM-R AsPC-1 cells, respectively. Among GEM-S and GEM-R PANC-1 cells, kaempferol reduced migration by 70.1% and 66.0% (both *p* < 2 × 10^−16^), respectively.

### 3.7. Effect of GEM Resistance and MMP-1 Knockdown on the Invasion of PaCa Cells

To evaluate changes in invasive potential following MMP-1 knockdown in PaCa cell lines, we performed a Matrigel invasion assay, similar to the Transwell migration assay. In both MIA PaCa-2 and SW1990 cell lines, GEM-R cells exhibited enhanced invasion compared with their respective GEM-S cells.

**Figure 9:**
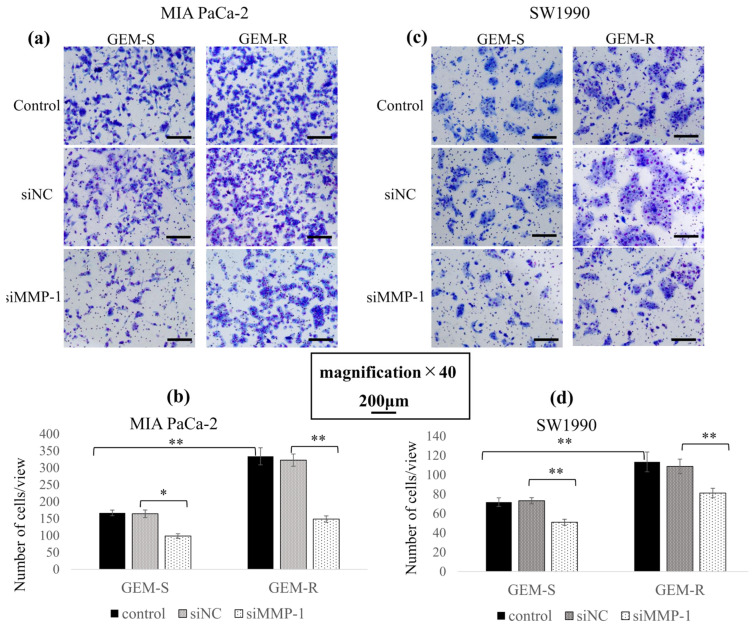
Effects of MMP-1 knockdown on invasive ability assessed by Matrigel invasion assay. Control, siNC, or siMMP-1 PaCa cells (1 × 10^5^) were seeded into Matrigel-coated upper chambers and cultured for 24 h. Invaded cells were fixed, stained, and imaged (**a**,**c**). Magnification: ×40; scale bar: 200 μm. Cells from nine random fields were counted (**b**,**d**). Comparisons were assessed using one-way ANOVA followed by Bonferroni’s test. Data are presented as the mean ± SD. * *p* < 0.05; ** *p* < 0.01.

Compared with the siNC-transfected cells, A significant reduction in invading cell numbers was observed in both cell lines after MMP-1 knockdown. In MIA PaCa-2 cells, the number of invading MMP-1-knockdown cells was decreased by 40.1% (*p* = 0.00000003) in GEM-S cells and by 54.0% (*p* < 2 × 10^−16^) in GEM-R cells (Figure 9a,b). In SW1990 cells, MMP-1 knockdown reduced the number of invading cells by 31.3% (*p* = 6.4 × 10^−7^) in GEM-S cells and by 25.4% (*p* = 2.6 × 10^−9^) in GEM-R cells (Figure 9c,d).

These findings indicate that MMP-1 contributes to the enhanced invasion observed in GEM-R PaCa cells.

### 3.8. Effect of Kaempferol on the IL-1β-Induced Invasion of PaCa Cells

Following the invasion assay under MMP-1 knockdown conditions, we next examined changes in the invasion ability of PaCa cells after treatment with kaempferol or the MMP inhibitor batimastat. Consistent with the Transwell migration assay, treatment with 50 µM kaempferol or 400 ng/mL batimastat reduced the number of invading cells. IL-1β increased the invasion ability of PaCa cells, and both kaempferol and batimastat effectively inhibited this increase. In MIA PaCa-2 cells, kaempferol and batimastat reduced the number of invading GEM-S cells by 73.6% (*p* = 1.6 × 10^−13^) and 74.5% (*p* = 2.2 × 10^−12^), and the number of GEM-R cells by 68.9% and 82.0% (both *p* < 2.6 × 10^−16^), respectively. In SW1990 cells, kaempferol and batimastat decreased invasion by 70.5% (*p* = 5.0 × 10^−7^) and 57.6% (*p* = 1.5 × 10^−7^) in GEM-S cells and by 74.2% (*p* = 1.5 × 10^−9^) and 68.0% (*p* = 2.0 × 10^−11^) in GEM-R cells, respectively.

**Figure 10:**
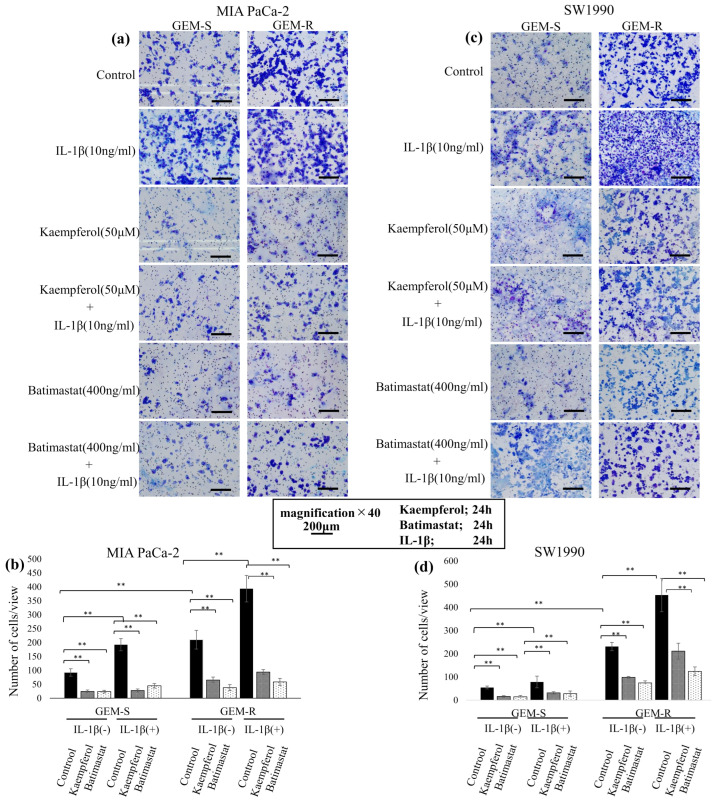
Effects of kaempferol and batimastat on invasive ability of PaCa cells. Invasion assays were conducted as in Figure 9. PaCa cells (1 × 10^5^) were treated with IL-1β, kaempferol, or batimastat for 24 h. Invaded cells were fixed, stained, and imaged (**a**,**c**). Magnification: ×40; scale bar: 200 μm. Cell counts from nine random fields are shown (**b**,**d**). Comparisons were assessed using one-way ANOVA followed by Bonferroni’s test. Data are presented as the mean ± SD.; ** *p* < 0.01.

Kaempferol and batimastat also suppressed the IL-1β-induced increase in invasion. In MIA PaCa-2 cells, kaempferol reduced IL-1β-enhanced invasion by 85.5% (*p* < 2 × 10^−16^) in GEM-S cells and 76.2% (*p* < 2 × 10^−16^) in GEM-R cells, while batimastat reduced invasion by 76.8% (*p* < 2 × 10^−16^) in GEM-S and 85.2% (*p* < 2 × 10^−16^) in GEM-R cells. In SW1990 cells, kaempferol reduced IL-1β-stimulated invasion by 60.3% (*p* = 1.2 × 10^−9^) in GEM-S cells and 53.4% (*p* < 2 × 10^−16^) in GEM-R cells, whereas batimastat reduced invasion by 64.2% (*p* = 2 × 10^−10^) in GEM-S cells and 72.6% (*p* < 2 × 10^−16^) in GEM-R cells, respectively (Figure 10a–d).

These findings suggest that kaempferol effectively suppresses both the baseline and IL-1β-enhanced invasion ability of PaCa cells, comparable with the effect of the MMP inhibitor batimastat.

### 3.9. The Role of Phosphorylated Akt and p65 Activity in the Altered MMP-1 Protein Expression in PaCa

To evaluate the signaling pathways involved in the inhibition of MMP-1 by kaempferol, we first analyzed MMP-1 and phosphorylated Akt (p-Akt) levels in PaCa cell lines by Western blotting. Treatment with 50 µM kaempferol suppressed the levels of both MMP-1 and p-Akt in GEM-S and GEM-R MIA PaCa-2 cells and SW1990 cells. IL-1β treatment increased MMP-1 and p-Akt levels in MIA PaCa-2 cells, while no significant differences were detected in SW1990 cells compared with the control. Kaempferol attenuated the IL-1β-induced increases in both MMP-1 and p-Akt expression levels.

**Figure 11:**
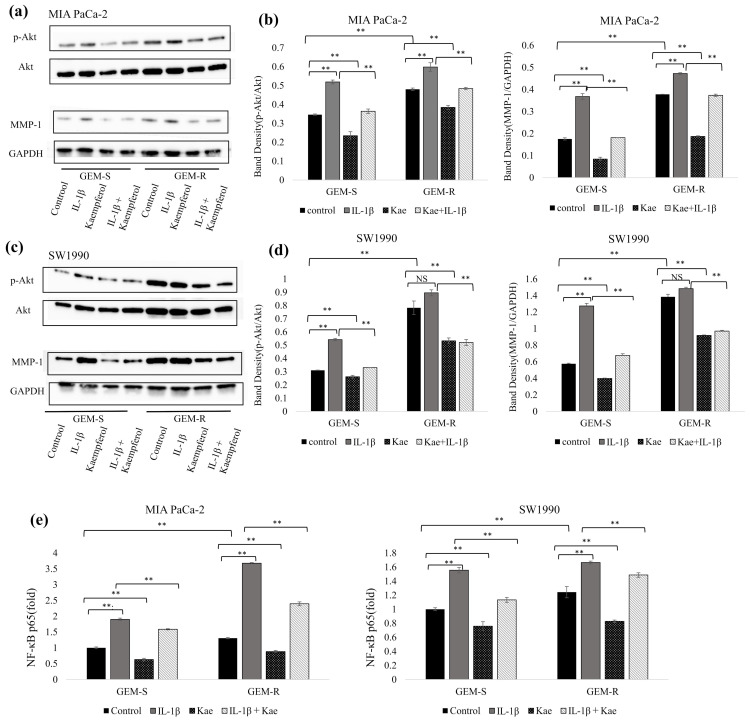
Effects of kaempferol on MMP-1, p-Akt, and NF-κB activity. (**a**,**c**) MMP-1 and p-Akt protein levels were assessed by Western blotting. (**b**,**d**) Band densities were normalized to GAPDH and total Akt, respectively. (**e**) NF-κB p65 activity in nuclear extracts was measured using a TransAM NF-κB p65 kit. Kaempferol was applied for 24 h; IL-1β was added 30 min before the end of incubation. Comparisons were assessed using one-way ANOVA followed by Bonferroni’s test. Data are presented as the mean ± SD. *n* = 4; ** *p* < 0.01.

Next, we tested the effects of kaempferol on MMP-1 and p-Akt expression levels in the PaCa cells. In MIA PaCa-2 cells, kaempferol reduced MMP-1 and p-Akt expression levels by 52.0% (*p* = 2.2 × 10^−9^) and 32.4% (*p* = 8.0 × 10^−6^) in GEM-S cells and by 50.5% (*p* = 1.2 × 10^−14^) and 20.0% (*p* = 5.8 × 10^−5^) in GEM-R cells, respectively. Kaempferol also suppressed the IL-1β-enhanced expression of MMP-1 and p-Akt by 50.7% (*p* = 1.1 × 10^−14^) and 29.9% (*p* = 9.0 × 10^−8^) in GEM-S cells and by 21.1% (*p* = 2.8 × 10^−10^) and 19.1% (*p* = 6.3 × 10^−6^) in GEM-R cells, respectively (Figure 11a,b). In SW1990 cells, kaempferol reduced MMP-1 and p-Akt levels by 30.6% (*p* = 9.4 × 10^−6^) and 15.8% (*p* = 0.00018) in GEM-S cells and 33.8% (*p* = 5.3 × 10^−12^) and 32.0% (*p* = 1.4 × 10^−8^) in GEM-R cells, respectively (Figure 11c,d).

Next, nuclear extracts were prepared following the kaempferol and IL-1β treatments, and NF-κB p65 activity was evaluated using the TransAM NF-κB p65 assay. In both cell lines, p65 activity was higher in GEM-R than in GEM-S cells. Kaempferol significantly decreased p65 activity. In MIA PaCa-2 cells, kaempferol reduced p65 activity by 37.1% (*p* = 0.0019) in GEM-S cells and 32.5% (*p* = 0.0007) in GEM-R cells compared with the control. Kaempferol also suppressed IL-1β-induced p65 activation by 16.5% (*p* = 0.0062) in GEM-S cells and 34.9% (*p* = 5.2 × 10^−11^) in GEM-R (*p* = 2.6 × 10^−8^) cells. In SW1990 cells, p65 activity was reduced by 24.1% in GEM-S cells and 33.4% in GEM-R cells following kaempferol treatment, and the IL-1β-enhanced p65 activity was decreased by 27.1% in GEM-S (*p* = 1.1 × 10^−8^) cells and 10.7% in GEM-R cells (Figure 11e).

These results indicate that kaempferol suppresses MMP-1 expression in PaCa partly via inhibition of Akt phosphorylation and NF-κB p65 activation.

## 4. Discussion

Among the MMP family members, MMP-2 and MMP-9 have traditionally received the most attention; however, MMP-1 is another member that exhibits strong clinical relevance. Poor prognosis in PaCa patients has been linked to increased MMP-1 expression [11,12,13]. Zhou et al. reported that the MMP1-1607 (1G > 2G) polymorphism is correlated with a higher risk of cancer development [14], and Kaplan–Meier analyses indicated significantly reduced overall survival and recurrence-free survival in PaCa patients with high MMP-1 expression. The serum MMP-1 level has been correlated with clinical stage and lymphatic metastasis in PaCa [15]. Furthermore, MMP-1 has been implicated in perineural invasion [16], and Chen et al. demonstrated that suppressing MMP-1 reduces PaCa cell migratory and invasive capacities and limits metastatic progression in vitro as well as in vivo [17].

While batimastat [18], marimastat [19], and other broad-spectrum MMP inhibitors, which act as zinc ion chelators and inhibit MMP activity [19], have advanced to clinical trials, their efficacy has remained suboptimal. The major reasons for these failures include insufficient inhibitor specificity and inadequate understanding of the complex tumor biology underlying MMP-related pathways [20].

Kaempferol belongs to the flavonoid family and is widely distributed. In addition to its anti-inflammatory [10], antibacterial [21], antioxidant [22], osteogenic [23], and neuroprotective properties [24], its anticancer activity has attracted considerable attention. Epidemiological studies indicate a potential inverse association between flavonoid-rich dietary intake, including kaempferol, and PaCa risk, particularly among smokers [25,26]. Other than PaCa, kaempferol demonstrates anticancer effects in gastric [27], colorectal [4,28], bladder [29,30], prostate [30], ovarian [31], and breast [32,33] cancers. In colorectal cancer, kaempferol suppresses migration and invasion by inhibiting MMPs [4]. In PaCa, previous studies have shown that kaempferol promotes apoptosis [34,35,36] while inhibiting proliferation and migration [37]; however, its effects on migration and invasion as mediated by MMP-1 suppression, especially under GEM-R conditions, have not been investigated. Additionally, although several reports highlight the involvement of MMP-1 in PaCa progression, altered MMP-1 expression after GEM resistance development, and the consequences of MMP-1 suppression have not been fully elucidated.

In the present study, we demonstrated that PaCa cells show higher MMP-1 expression and further elevated in GEM-R PaCa cells. Kaempferol reduced MMP-1 expression and suppressed migration and invasion even in GEM-R PaCa cells, accompanied by inhibition of Akt phosphorylation and NF-κB p65 activity. Supporting our findings, previous reports have shown that kaempferol modulates key oncogenic pathways. Lee et al. reported that kaempferol induces apoptosis by downregulating the Src/Akt/ERK pathway via EGFR inhibition [37]. Wang et al. showed that kaempferol induces apoptosis by inhibiting the Akt/mTOR pathway via TGM-2 [36], while other studies reported that kaempferol inhibits the STAT3 pathway by increasing reactive oxygen species production and decreasing SHP-1 expression [34]. Interestingly, kaempferol exhibited antioxidant properties in a neuroprotection study [22], yet induced oxidative stress to exert anticancer effects in PaCa [34,36]. NF-κB, which interacts with the Akt/mTOR pathway, regulates MMP expression [38]. We previously reported that NF-κB contributed to GEM resistance in PaCa [3,39]. The present study supports this relationship by showing increased MMP-1 expression via Akt and NF-κB signaling in GEM-R PaCa, an effect that was effectively suppressed by kaempferol.

In colorectal cancer, kaempferol reduced Akt/mTOR phosphorylation and suppressed MMP-1, MMP-2, and MMP-9 kaempferol inhibited migration and invasion [4]. However, that study used kaempferol at its IC_50_ concentration, and the cytotoxicity potentially induced by this concentration may confound motility assessments. In contrast, Kai et al. reported that MMP-1 knockdown suppressed PI3K/Akt/c-Myc pathway activation in colorectal cancer [28]. Similarly, in breast cancer, increased MMP-1 expression activated NF-κB signaling, promoting epithelial–mesenchymal transition [33]. Those findings suggest that similar mechanisms likely occur in PaCa, in which kaempferol may suppress MMP-1 expression via pathways involving reactive oxygen species generation, PI3K/Akt/mTOR signaling, NF-κB signaling, and STAT3 inhibition. In addition, reduced MMP-1 expressions may further downregulate Akt/mTOR signaling, potentially creating a positive regulatory loop. Following this, NF-κB, which is in crosstalk with Akt signaling, may likewise exhibit reduced activity as a result of MMP-1 suppression. Future studies will verify this possibility using specific inhibitors and rescue experiments.

Moreover, kaempferol has been reported to form a 1:1 complex with zinc ions [40]; that is, the zinc-dependent nature of MMP activity raises the possibility that kaempferol may interfere with MMP function through metal chelation; thus, kaempferol is suggested to inhibit MMP-1 not only through signaling pathways but also via direct mechanisms.

Currently, standard treatments for pancreatic cancer include regimens such as gemcitabine (GEM) combined with nab-paclitaxel (nab-PTX) or FOLFIRINOX [41,42]. The GEM + nab-PTX regimen has demonstrated an overall survival (OS) of 8.5 months, compared to 6.7 months with GEM alone, indicating superior therapeutic efficacy [41]. FOLFIRINOX has shown an OS of 11.1 months and progression-free survival (PFS) of 6.4 months, both of which are improvements over GEM alone (OS: 6.8 months; PFS: 3.3 months); however, its use is generally limited to patients with good performance status [42]. Immune checkpoint inhibitors (ICIs) have advanced in the treatment of various malignancies in recent years; however, their monotherapy efficacy in pancreatic cancer remains limited due to the tumor’s immune microenvironment [43]. Thus, identifying predictive biomarkers of ICI efficacy and developing effective combination therapies are needed. Given this situation, GEM remains one of the most crucial agents in pancreatic cancer treatment. Future studies are planned to evaluate the combinatory effects of kaempferol with GEM-based chemotherapy and other chemotherapeutic regimens. Although the combinational effects of kaempferol and gemcitabine (GEM) were not examined in this study, Denda et al. [3] demonstrated that flavonoids are capable of resensitizing GEM-resistant cells. Therefore, future studies will investigate the combined treatment of kaempferol and GEM, focusing on cell viability, migration, and invasion. As reported by Barve et al. [44], the bioavailability of kaempferol is low and is estimated to be less than 2%. Assuming a bioavailability of 2%, achieving a blood concentration of 50 μM (50 × 10^−6^ mol/L) would require 716 mg/L of kaempferol per liter of circulating blood (calculated as molecular weight × 50 × 10^−6^ g/L; 286 × 50 × 10^−6^ × 2%). Given that the circulating blood volume of a 60 kg adult male is approximately 4000 mL, the required dose would be 2860 mg (48 mg/kg). According to DuPont et al. [45], the plasma concentration of kaempferol reaches its maximum approximately 6 h after oral administration, declines to half of the peak level at around 12 h, and decreases to about 20% after 24 h. From the perspective of the half-life, a dosing interval of approximately 24 h would therefore be appropriate. However, evaluation based on the area under the curve (AUC) is also important, and in vivo studies using lower doses of kaempferol than those previously reported are warranted. With regard to the dosing period, previous animal studies have administered kaempferol for 4–6 weeks [29,36]; if kaempferol were to be used clinically for antitumor purposes, an observation period of at least several months, similar to other chemotherapeutic regimens, would be considered necessary.

Several limitations must be acknowledged. First, the signaling pathways by which kaempferol suppresses MMP-1 remain incompletely understood. If, as reported in colorectal cancer [28], MMP-1 inhibition is found to attenuate Akt/mTOR phosphorylation in PaCa, the therapeutic potential of kaempferol would be even greater due to its secondary effects.

Second, while NF-κB is known to regulate MMP-1 transcription [38], our study demonstrated only the concurrent inhibition of NF-κB activity, Akt phosphorylation, and MMP-1 protein expression by kaempferol; thus, elucidating whether kaempferol directly affects MMP-1 mRNA expression remains an important issue for future research, and we will perform these experiments in the near future. In breast cancer, MMP-1 has been reported to promote epithelial–mesenchymal transition (EMT) through the NF-κB signaling pathway [33]. In future studies, we plan to analyze EMT markers such as E-cadherin, *N*-cadherin, vimentin, and Snail to determine whether a similar phenomenon occurs in pancreatic cancer. In addition, kaempferol has poor bioavailability, reportedly below 2% [44], which is a major challenge for clinical application. Strategies such as nanoparticle formulation [46], complex formation with hydroxypropyl-β-cyclodextrin, and liposomal encapsulation [47] may improve absorption. To date, kaempferol has been shown to exert antitumor effects in vivo in pancreatic cancer and other cancer types [29,36], and MMP-1 has been demonstrated to regulate invasion and metastasis of pancreatic cancer in vivo [17]; however, future studies are needed to verify whether the MMP-1 inhibitory effect of kaempferol is achievable in vivo and to explore methods to enhance its bioavailability. We plan to conduct further evaluations in gemcitabine-resistant pancreatic cancer xenograft and orthotopic models. The present study employed GEM-S and GEM-R variants of MIA PaCa-2 and SW1990 pancreatic cancer cell lines similar experiments using AsPC-1 and PANC-1 will be conducted in future studies to validate the robustness of our results.

In conclusion, this study is the first to report altered MMP-1 expression in GEM-R PaCa and the therapeutic potential of kaempferol for GEM-R disease. GEM-R PaCa exhibited increased MMP-1 expression, contributing to enhanced migration and invasion. Kaempferol suppressed MMP-1 expression via inhibition of the Akt and NF-κB pathways, thereby reducing the migration and invasion capacities of GEM-R PaCa cells, similar to the established effects of the MMP inhibitor batimastat. These findings suggest MMP-1 to be a promising target for treatment in GEM-R PaCa, and kaempferol may represent a novel therapeutic approach for GEM-R PaCa by targeting MMP-1.

## 5. Conclusions

MMP-1 expression is significantly increased in PaCa cells relative to normal pancreatic ductal epithelial cells and becomes further upregulated as gemcitabine resistance is acquired, contributing to enhanced migration and invasion capacities. Kaempferol suppresses this process at non-cytotoxic concentrations, exerting clear antitumor effects through MMP-1 inhibition. Based on these findings, kaempferol emerges as a potential MMP-1-targeted therapeutic candidate for PaCa that may offer fewer adverse effects than current chemotherapeutic options.

## Data Availability

The data presented in this study are available from the corresponding author upon request. The data are not publicly available due to privacy.

## Supplementary Materials

The following supporting information can be downloaded at: https://www.mdpi.com/article/10.3390/nu18030380/s1, Figure S1: Effects of kaempferol on migration in GEM-S/GEM-R AsPC-1 and PANC-1 assessed by Transwell assay. PaCa cells (1 × 10^5^) were treated with kaempferol for 24 h and subjected to migration assays similar to Figure 5 and Figure 7. Migrated cells were fixed, stained, and imaged (**a**,**c**). Magnification: ×40; scale bar: 200 μm. Quantification was performed from nine random fields (**b**,**d**). Comparisons were assessed using one-way ANOVA with Bonferroni’s test. Data are presented as the mean ± SD. ** *p* < 0.01.
