# Supplementary figures and images for: Kaempferol Inhibits MMP-1-Mediated Migration and Invasion in Gemcitabine-Resistant Pancreatic Cancer Cells
Source: Nutrients. 2026 Jan 23;18(3):380. doi: 10.3390/nu18030380 (PMC12899928; doi:10.3390/nu18030380)

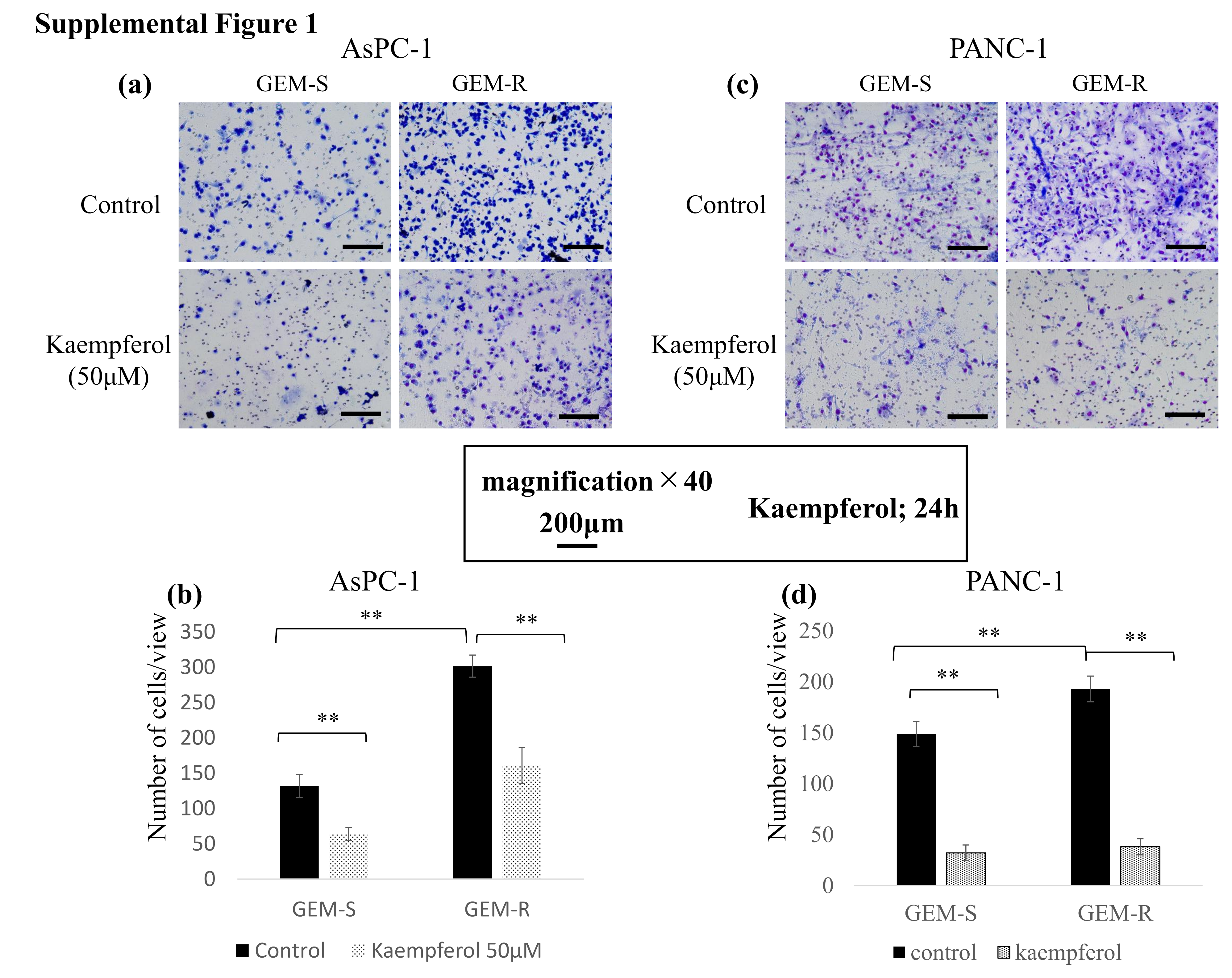

Supplement: Supplementary file 1 [file nutrients-18-00380-s001.zip › nutrients-4085944-Figure S1.tiff]
